# Global competency impact of sustained remote international engagement for students

**DOI:** 10.1186/s12909-023-04333-x

**Published:** 2023-06-12

**Authors:** Tracy Kelly, Abebe Bekele, Sonja G. Kapadia, Simrat K. Jassal, Darlene Ineza, Theogene Uwizeyimana, Olivia Clarke, Tabor E. Flickinger, Rebecca Dillingham, Marcel E. Durieux

**Affiliations:** 1 University of Virginia, Charlottesville, USA; 2 University of Global Health Equity, Kigali, Rwanda

**Keywords:** Remote engagement, Cultural competency, Sustainability, Global partnership, Mixed method

## Abstract

**Background:**

To provide just equity in academic exchange, as well as to reduce prohibitive travel cost and address environmental concerns, the past paradigm of international student exchange has fundamentally shifted from one directional travel to mutually beneficial bidirectional remote communication between students all over the globe. Current analysis aims to quantify cultural competency and evaluate academic outcomes.

**Methods:**

Sixty students half from the US and half from Rwanda grouped in teams of 4 engaged in a nine-month project-focused relationship. Cultural competency was evaluated prior to project initiation and six months after completion of the project. Student perspective of project development was analyzed weekly and final academic outcome was evaluated.

**Results:**

Change in cultural competency was not significant; however, students did identify satisfaction in team interaction and academic outcomes were achieved.

**Conclusion:**

A single remote exchange between students in two countries may not be transformative but it can provide cultural enrichment and successful academic project outcome and may serve to enhance cultural curiosity.

## Introduction

Universities worldwide recognize the importance of global awareness as a life skill for their students and are developing educational programs to enhance cultural competency. The concept of cultural competency in academics is relatively new and yet to be fully actualized as a discipline. Diversity, cultural quotient, and resilience all may be used to define a sensitivity and awareness of divergent values, customs, and beliefs different from one’s own. Students in well-resourced countries often travel abroad for academic study, acquisition of language skills, research endeavors and cultural enrichment. However, unidirectional travel by students from high income countries inherently creates a state of inequity and a lack of reciprocity with the host country. Inequity is further compounded when the program is designed and supervised by the sending university with little or no input from the receiving institution, exemplifying long standing Western colonialist practices. To further exaggerate exclusivity, programs are often more easily accessible to privileged students at a university while others with full academic schedules, financial constraints and/or family obligations are unable to travel. For example, while students of color make up 40% of graduating students, they form only a quarter of those who travel abroad [1]. Furthermore, the carbon footprint of air transportation and its impact on climate change causes many students to be dubious of frivolous international travel. Lastly, the SARS-CoV-2 pandemic and requisite travel bans elevated interest of global partners to consider alternative academic engagement.

Interest has therefore grown in pursuing other means of international education, particularly with the ability of technology to form bi-directional relationships with students throughout the world. Such programs should not be implemented just because they can, but only when effectiveness is demonstrated. However, scant data exists to assess the effectiveness of virtual international engagement and its impact on global competency. The University of Virginia (UVA) in the US and the University of Global Health Equity (UGHE) in Rwanda therefore set out to assess, through a mixed methods approach, the effect on cultural competency and perceptions of a long-term remote international engagement for students.

## Review of the literature

Interest in international student engagement has escalated in the last decade due to increased global awareness, ease of travel, interest in post-graduation global employment, and the evolving concept of global citizenship. In 2018/2019 close to 250,000 US students traveled abroad, more than half of them to destinations in Europe [2]. Because of this rapid escalation, many international programs are poorly designed, unsupervised, unsustainable, and can push ethnocentric perspectives with little benefit to the host country [3–5]. Many students travel for short amounts of time, and research shows that, for example, two weeks is insufficient to properly achieve course objectives [6]. Moreover, students require considerable time to establish themselves in their new environment before true cross-cultural learning can begin, thus consuming a significant portion of the exchange duration and delaying the learning process. Many international programs have verified course objectives, but these are often not shared with host country preceptors [7]. Therefore, objectives may not be encouraged or even supported by the host country itself [8].

Recent attention has turned to the escalating financial and environmental cost of international educational programs. Logistical details for traveling students (i.e., flight arrangements, medical insurance, immunization, visas, and language preparation) is a highly involved process both in terms of personnel labor and financial costs [9]. More daunting is the impact air travel places on individual carbon footprint. Universities therefore are looking for creative and responsible solutions such as remote programs to reduce global carbon footprints [10].

Collaborative online learning programs have existed for many years and amplified since the recent worldwide pandemic [11]. While virtual experiences cannot duplicate physical immersion programs and it may take several iterations of bi-directional exchange to verify measurable impact, the benefit of alternative collaboration is obvious in the current digital world. The longstanding bias of unidirectional travel is removed in remote exchange, yet standards of bi-directional engagement, such as set forth by the Consortium of Universities of Global Health [12, 13] should be followed regardless of if the exchange is actual or remote. Research, however, is nascent in providing evidence and guidance for remote global exchange suggesting satisfaction but drawbacks as well. On one hand, they can enhance student learning and promote global competency. On the other hand, unique learning platforms, limited bandwidth, inconsistent internet access, and time zone discrepancy can inhibit student to student interaction [14, 15]. The benefits and challenges of such programs must be assessed to define their place in the effort to promote global competency. Cultural aptitude measuring tools are available: [16–19] however, research on increasing cultural competency via remote exchange format for both parties is limited [20].

The aim of this study therefore was to evaluate a goal-oriented bi-directional learning experience, named eGlobal, designed to connect students in two countries, the United States and Rwanda. The eGlobal goals were to provide students with advancement in cultural competency, progressive group dynamics, enriching cultural exchange, gratifying encounters, and successful project outcomes. The evaluation investigated to what extent the program had met these goals.

## Methodology

Using a mixed method approach, we performed a prospective single-cohort study of the eGlobal program. The evaluation included assessment of participants’ cultural competency and the quality of their experience in the program.

### Program description

The University of Virginia (UVA) Center for Global Health Equity in the United States has had an ongoing partnership with the University of Global Health Equity (UGHE) in Rwanda, with up to 20 UVA students involved in six-week internships each year. Acknowledging the obvious inequity in continued unidirectional travel for UVA students without reciprocal travel for UGHE students and attempting to offset the historic imbalance of such programs was a driving force behind this study. Building on this existing partnership, a convenience sample of interested student subjects were approached to participate in a nine-month remote engagement with students from UGHE. Additional student subjects were gathered using a snowball approach. UGHE encouraged their entire second year Bachelor of Science / Bachelor of Surgery (MBBS) cohort to join the program. In total, 30 students from UGHE and 30 students from UVA were recruited for participation in an experimental virtual international engagement. Small research projects were written by the research team (see Appendix 1), with topics designed to promote cross-cultural discussion and that could be completed without the collection of new data. Based on their interest in the project focus, teams of four students were formed – two UGHE students and two UVA students per team. Weekly team engagement of about 1 h to work on the research project occurred via WhatsApp or Google platforms. Individual students were expected to spend about 1 other hour during the week on research. The study team explicitly encouraged casual conversations too among student teams regarding cultural rituals, holiday celebrations, family tradition and local weather, even encouraging video chats of each other’s schools to offer original ways for cultural exchange.

Ethics Institutional Review Board approval was obtained at both participating institutions in Virginia and Rwanda. This investigation conforms with the principles outlined in the Declaration of Helsinki. After oral and written explanation of the study process and goals were explained, all study participants gave written informed consent to participate in the study. Compensation of $650 for participation was provided to each student after completion of all aspects of the study. At UGHE, this payment was applied directly to the students’ education accounts. At UVA, this payment was directly deposited into the students’ portals. A pilot study of one team began their engagement two months earlier than the subsequent teams to identify any significant obstacles and make appropriate modifications as needed. A four-hour orientation was provided to all students on the aims of the study, history and culture of their international counterparts, and subject responsibility. Project mentors, faculty members from both universities with expertise in subject matter and global programming, were assigned to each of the 15 projects to offer direction, expertise, and encouragement. To support the students, an administrator at both universities was assigned to remind student subjects of assignment goals and deadlines and to troubleshoot any challenges.

At the completion of the nine months a remote research symposium was held where each team presented their project findings. In addition, the editors of Conflux, the UVA global health research journal which publishes original student research, agreed to publish submitted papers of each project team in a special issue [21].

### Cultural competency

A selection of cultural aptitude tools was evaluated for use in this study to compare changes in intercultural development over the study period. Several tools were eliminated due to self-appraisal bias, pre and post-test exposure bias and difficulty of international availability (e.g., Global Perspective Inventory -GPI, Intercultural Development Inventory—IDI, Program for International Student Assessment / Organization for Economic Cooperation and Development—PISA OECD). Ultimately, the Global Competence Aptitude Assessment (GCAA) was selected [19]. For context, the GCAA was introduced in 2009 after undergoing extensive research about its validity and reliability and was revalidated in 2017. It has been used in 115 countries across six continents and is currently used in academic, business and government sectors [22]. The GCAA is a self-assessment 80 item tool that measures a participant’s level of global competence evaluating both internal (self-awareness and attitudes) and external (cultural knowledge and interpersonal skills) readiness for global interactions and has specific academic application in measuring student achievement of learning outcomes prior to and after interventions. There is cross referencing throughout the tool using assorted styles of questions and varying degrees of difficulty to ensure comprehensive measurement. To compare pre- and post-intervention knowledge, attitude, and behavior changes, the GCAA was administered to all 60 students at the start of the orientation (pre-intervention and six months after completion of all engagement (post-intervention). The six-month delay was designed to provide sufficient reflection time after the program's completion.

### Data collection and analysis

For the duration of the program, each student submitted a weekly assignment (WA) detailing group and individual hours of engagement and the nature of their experience that week. The WAs consisted of six prompts with an optional document upload feature for pictures or other documents (Appendix 2). This ensured uniform data collection methodology and provided consistent self-reflection for the students. The continuous feedback provided intimate, instantaneous snapshots into students’ experiences in eGlobal on a weekly basis. The logistical coordinators monitored the WAs each week for urgent issues and to assess participation. Using Dedoose qualitative analysis software and Microsoft Excel the responses were analyzed into a qualitative coding scheme, which was developed inductively by the logistical coordinators. This codebook was refined iteratively using constant comparisons methodology until thematic saturation was achieved. (Appendix 3). Administrative staff at both institutions determined coding themes independently initially, then together to resolve discrepancies. A further review was completed independently by another staff member with final codes determined by consensus. To safeguard data quality a final submission to expert reviewers establishing inter-rater reliability. The coding scheme consisted of parent codes (e.g., “Challenges”) and one level of associated sub-codes to identify more specific characteristics within each parent code. Each code was applied only once per excerpt, but excerpts could have more than one code applied if multiple themes were present. Also, codes could be applied more than once per participant if they were relevant to multiple separate ideas or events of the week. The entire data set of weekly responses was coded, so that code frequencies could be determined. For all participant quotes in this article, any potentially identifying information, such as names, was redacted for privacy. Otherwise, all original syntax and spelling are unedited, to preserve participant voice.

The validity of the coding scheme was checked by independent assessment of relevant themes by the logistical coordinators and a senior researcher involved with the eGlobal program, as well as by an unaffiliated researcher with no direct involvement with the program. After the coding process was completed, the data was analyzed to assess for correspondence of the themes derived from participants’ experiences with the goals of the program. Specifically, the analysis focused on themes related to intercultural interaction, group dynamics, and project progress/outcomes.

## Results

### Participant characteristics

Students at UGHE, located in Butaro, Rwanda, were all in the second year of their MBBS program, a six-year dual degree Bachelor of Medicine and Masters of Global Health. The University of Virginia students had more diverse academic backgrounds with some enrolled in the undergraduate college, others in the graduate nursing and medical school program. The ethnic background and travel experiences of the cohorts likewise were divergent. All the UGHE were Rwandan citizens, sharing the same native language, and none had the opportunity to travel outside of their home country. UVA students were ethnically diverse speaking several different languages many having extensive global travel. Of the 60 initially participating students, 59 completed the study. One UVA student withdrew from the study four months into the project for personal reasons. In total 15 projects were presented at the symposium and 15 papers were published in the UVA Conflux journal [21].

### Cultural competency

Pre- and post-intervention GCAA survey results are provided in Appendix 4. The GCAA assessment indicated a stronger internal and external readiness in all fields for the UVA students over the Rwandan students prior to the intervention, most notably in historical events and geographic familiarity and culture. This remained true six months after the intervention. In the UVA group pre- and post-intervention scores remained relatively flat in the internal readiness category with a slight reduction in score in several categories of external readiness. In the UGHE participants pre-and post-intervention scores also were not changed significantly, although analysis of scores do suggest deeper insight in some dimensions of external readiness.

### Program experience

Initially, time differences created a challenge to finding a convenient meeting time as Rwanda is seven hours ahead of Virginia. Most of the teams agreed that afternoon Central African Time (GMT + 2) / morning Eastern Daylight Time (GMT –4) worked best for their weekly meeting. Similarly finding a mutual platform was necessary. The most common means of communication used by the student teams was WhatsApp with Zoom and Email as less common alternative. Over the entire nine-month period the weekly hours of engagement, combining both group interaction and independent project work for over 80% of respondents, was about two hours per participant. On some weeks, especially during writing of the Conflux paper, independent work alone flexed above two hours.

#### Most prevalent themes

The students’ weekly reports demonstrated that the most common characteristics of their program experience fell into the categories of culture, group dynamics, meeting details, communication, project progress, challenges, and setbacks. Table 1 shows the top three subcodes within each category, in terms of greatest code frequency*.*

**Table 1:** Student program experience theme and sub-categories by frequency

**Main categories** Subcategories	**Example of Student Comments**	**Frequency** N % (#)
**Culture**	*“It is a significant achievement we made of being able to talk to each, share how the week went or what was interesting in that week, and more other fun activities we enjoyed.”*	**100% (766)**
1. College	*“We discussed the following topic:—what we would study if we were not studying our current majors—How we chose majors in our country, medical school, and scholarship—favorite artist and songs—Languages in our country (how many we study, when, what is the priority one)—we had fun!”*	35% (265)
2.Daily life	*“I can say that I know a little about my colleagues but as days come we will know each other better.”*	23% (177)
3. Holidays	*“We have discussed a variety of different cultural aspects including our diets and daily routine, what we do with our free time, and holidays like Halloween and Thanksgiving that are not celebrated in Rwanda.”*	13% (102)
**Group dynamics**	*“I didn't realize how easy it would be to build and keep up a relationship with people who live abroad and who you haven't met in person before. This project has truly shown how small the world is.”*	**100% (1062)**
1. Feeling more familiar with group	*“eGlobal has definitely changed my perspectives of relationships abroad. At first I was a bit skeptical as to how I would be able to build a bond with someone else if I never got to meet them in person, but meeting every week over has helped us build relationships that I did not think were feasible.”*	32% (344)
2. Good communication	*“I didn't realize that it could be so easy to develop a relationship with someone abroad. We bond so easily and it feels so normal to me like the distance doesn't make a difference.”*	19% (199)
3. Having fun	*“We have developed a genuine friendship and we share pictures and videos of what we did throughout the week as well as joke around.”*	17% (180)
**Meeting details**	*“I think my group excels in structuring the calls. We have a plan to work on the project every other week and use the alternative weeks to just converse. This provides a great way to separate the two main goals of this research project and allows us to focus on only one of the two every week.”*	**100% (170)**
1.Planning ahead to meet	*“We finally had a meeting without any technology issues and it was great to be able to talk. We got to know each other more today and I am excited for what we planned to do next week.”*	74% (126)
2.Organized	*“We accomplished a lot on writing the paper and are much more organized in how we plan to move forward with the rest of the writing process”*	21% (35)
3.Able to find a time to meet	*“All members were present so we were really able to get to talk as a group and discuss a regular schedule for meetings.”*	12% (20)
**Project progress**	*“We have been in communication over email. Although stressful, getting together this paper has definitely tested our group and I believe strengthened our teamwork.”*	**100% (1112)**
1.Interaction with mentor	*“We met with our mentor and received a lot of useful insight that we will use for our first draft submission.”*	20% (227)
2.Discussing next steps	*“We were quite productive in our meeting. We each contributed equally to working through comments on our first proposal draft and shared thoughts about next steps.”*	11% (120)
3.Division of work	*“This week during our meeting, we delegated sections of our first draft to work on/revise and remained in communication throughout the week regarding final edits.”*	11% (118)
**Challenges**	*“We also were a little bit confused about what to do for the project at first, but we kind of realized that we could take it a little easier and spend more time getting to know each other before we jumped right in.”*	**100% (105)**
1.Meeting people for the first time	*“It was a bit slow at first starting to talk to each other and be more comfortable, but now I feel like I can talk to everyone in my group almost as easily as I can my friends here at UVA that I've known for a couple of years now.”*	24% (25)
2.Lack of guidance	*“I do wish there was a bit more direction in the development of the projects. I feel like we have a lot of autonomy and sometimes its been hard to make a clear schedule and set deadlines.”*	17% (18)
3.Not talking about social life	*“I also feel like cultural learning aspect hasn’t really been focused on too much because it seems much easier to collaborate on our project when we’re online, as opposed to chat and learn about one another’s countries.”*	16% (17)
**Setbacks**	*“So far I have had great learning experiences with my group. However, having a time difference of 7 h as well as other academic-related issues like exams and assignments made it challenging to find a suitable time for scheduling a meeting.”*	**100% (668)**
1.Not able to meet	*“We were unable to have a weekly call due to conflicts in group members' schedules. Instead we largely communicated over WhatsApp to make plans for writing our research proposal, contacting our mentor, and hopefully being able to meet soon.”*	45% (301)
2.Not everyone available to meet	*“There was an issue with the meeting planning so unfortunately, one of the members was unable to come to this meeting, but we still had a lot of fun.”*	19% (125)
3.Prior obligations	*“We have gotten to become pretty close, but it is difficult to meet for a long time because all of us have very busy schedules”*	15% (100)

#### Alignment with program goals

Analysis of the students’ weekly assignments revealed that their self-reported experiences corresponded to the stated goals of the eGlobal program. These goals were to provide students with advancement in cultural competency, progressive group dynamics, enriching cultural exchange, gratifying encounters, and successful project outcomes.

Regarding cultural competency and cultural exchange, students expressed that the eGlobal program gave them an opportunity to share lore, traditions, and customs, to improve mutual understanding. For example, one student stated:

*“We learn about each other’s lifestyle and culture”*

Another observed,

*“eGlobal has helped me to improve my communication skills and the way I relate with people from abroad”*

For some students, the program allowed them to overcome preconceived ideas of the difficulty of cross-cultural interaction, for example,

*“I didn't know that I'd talk to someone foreign and I'd be familiar immediately and most surprisingly online”*

Another attributed their change specifically to the program:

*“eGlobal has definitely changed my perspectives of relationships abroad. At first I was a bit skeptical as to how I would be able to build a bond with someone else if I never got to meet them in person but meeting every week [online] has helped us build relationships that I did not think were feasible” (Clarification in brackets is by the investigators.)*

Some groups found cultural exchange more difficult than others though, for example,

*“I'm thinking we need to incorporate more activities just to get to know one another and for cultural exchange, because we've started diving into our topic and I think that's taking up most of our time”*

Language could also be a barrier; as one stated,

*“conducting all discussions in English as my second language is challenging since almost on a daily basis my discussions with friends are in Kinyarwanda.”*

For progressive group dynamics, students described movement from unfamiliarity with each other to effective communication. Some students experienced positive group interaction right away, such as.

*“Our group dynamic is very fun and we have no problem holding a conversation.”*

Others had to overcome initial awkwardness that improved as time went on. For example, one student said,

*“Each week conversations got easier”*

another said,

*“The conversation is not forced or uncomfortable anymore”*

The concept of gratifying encounters was seen in students’ descriptions of taking pleasure in the encounters with their groups with the intention of conveying meaningful dialogue and friendship. For some students, this aspect of the program was the most valuable part of the experience, for example,

*“Getting to know them better is my favorite part of the project”*

Another said,

*“now we have developed a genuine friendship and we share pictures and videos of what we did throughout the week”*

There could be an overlap between gratifying encounters and cultural exchange, as students formed friendships with people from diverse backgrounds from themselves. As one stated,

*“we understand the differences in the way we live but we also are doing our best to connect with our similarities and our struggles because it makes it easier to get the conversation to flow”*

Successful project outcome as defined by a symposium presentation and publication of a paper by the team was also a goal of the program. Project progress was a frequent theme of students’ weekly reflections, including the logistics of meetings, working with the group, interacting with the mentors, and getting the work done. However, the projects were often appropriately regarded as a secondary goal, while the group interactions were primary, for example,

*“Sometimes I still am not sure if I should be doing more regarding the project- all we have done so far is read articles, discuss them and take some notes- but I do understand that this is more centered on relationship-building and I feel like we have succeeded in that.”*

## Discussion

Our results indicate that long-term online interaction between small groups of students from Africa and North America can promote benefits in several domains related to cultural competency.

Demographics of the two student groups could account for discrepant pre-intervention GCAA scores. The UGHE students were all born in Rwanda, and none had traveled outside of their home country. Conversely the UVA students came from diverse ethnic backgrounds and with international experiences. This distinction may account for differences in both dimension of the assessment. Distinct from other studies using the GCAA, data results were blinded from subjects and program administrators and was not used to interject programmatic changes.

Historical legacy may also explain components of the study results. Rwanda is still recovering from its massive genocide less than a generation ago. The roots of both its political history and ethnic strife are shared by colonial interference and complicity. Since the reconciliation after the 1994 genocide against the Tutsi, Rwandans pride themselves on being a monoculture. They share one indigenous language, Kinyarwanda. Much of the population lives in the country and the economy is still largely based on subsistence farming. The population is overwhelmingly Christian. National unity and pride trump diversity and cultural distinction as historically tribal identification led to violent strife. Conversely, self-confidence and individuality are considered an asset in American culture, and diversity is celebrated. According to one of the investigators (TU), Rwandans value community and are educated in this system. A tradition (Umuganda) calls all Rwandans together once each month to perform shared community work. Rwandans are naturally slow to reveal emotions and opinions, and this may affect their ability to welcome new cultural experiences. Perhaps wary of repeating previous situations by wealthy nations, students’ responses may be interpreted as cautious and guarded. Shared consensus dominates individual opinion. These phenomenon in homogeneity and group harmony may help to explain differences in GCAA results in the Rwandan participants, particularly related to open mindedness and individual awareness.

Finally, the completion of the survey for both groups ensured payment compensation and therefore attention to correctly answering post-intervention questions may have been less motivating.

Personal student to student global engagement offers value to the individual students in the form of global friendship and partnership in project goals. But the value goes beyond the student level and embraces bi-directional respect at the university level as well. Planning the project and subsequent pivoting of the study design allowed faculty at both universities to intersect on how to best meet study objectives. As a result, the relationship between the institutions was felt to be strengthened significantly by this program.

While the publication in the UVA global journal Conflux became an enticing goal, it also placed an additional burden on the students who were juggling academic assignments and end of year exams. Writing of the paper, rather than the group relationship, became a focal point during the second half of the project. One UVA student felt that superior writing skills by some members of the team created a false hegemony and jeopardized the trust and cohesiveness the group developed over the last nine months. To allay this concern a different format without an imposed paper such as monthly topic-driven conversations with a mentor-directed involvement could be considered to allow a deeper conversation on cultural differences and respectful communication.

### Limitations of the study

The most prominent limitation of this study is incomplete or biased feedback from students as analytics are only measured by code frequency and response quality. Moreover, results mirror only that of the students’ written responses. Additionally, code selection was left to the discretion of the three logistical coordinators who reviewed the data, thus introducing a degree of bias and error to the quantitative data.

Another limitation of this study is the absence of a control group who travel to Rwanda for an in-country experience. We recognize the limitation of this non-comparative study and initially planned to have such a group. COVID-19 restrictions made this impossible. Since several years passed and the pandemic introduced many new variables related to student international experiences, it seems unlikely that a valid comparison group is still a feasible proposition.

Finally, it is always conceivable that a study of this nature might suffer from additional biases, including implicit Global North and Western biases that were unaccounted for by the authors.

## Conclusions / future direction

While the program was designed and funded before the SARS-2 COV pandemic, the recruitment of subjects did coincide with movement towards distant learning and international travel restrictions. These changes may have made students more interested in a remote activity that they otherwise might not have considered. We have since continued the program as a voluntary (and unpaid) activity for students at UVA and UGHE, and still find enormous interest in participating. This indicates that a long-term engagement as offered by eGlobal fills an unmet need for students in both locations. We accept that one experience alone may not be transformative but may be a vehicle to motivate further cultural engagement. Several students who participated in the initial eGlobal experience signed up for the subsequent editions.

Individual cultural competencies are advanced through the interplay of personal experiences, international travel, and participation in cultural customs. There are obvious limitations to achieving these interactions through remote experiences. However, our results show the value of remote exchange as perceived by students in two vastly different countries. These findings suggest that long-term remote engagement might provide an important venue for international engagement, with benefits from academic, financial, and environmental viewpoints.

## Data Availability

The datasets generated and analyzed during the current study are available at this website https://docs.google.com/spreadsheets/d/1ux2y9M7hXGpajdj9S4TV1c7ZWlGnnnKq/edit#gid=1723545061.

## Appendix 1

Research projects for remote eGlobal group 2020 – 2021.

1. Community health workers in the US
2. Climate impact of humanitarian assistance
3. Global surgery
4. Road accidents
5. The opioid crisis
6. Feasibility of nurse practitioners in Rwanda
7. Use of social media for nursing education and research: a comparison of US vs Rwanda engagement
8. Condom Usage in Rural Areas
9. Sexual Violence and Healthcare
10. Telepsychiatry to Address Lack of Mental Health Resources
11. Vaccine Hesitation
12. Improving the sexual health of adolescents
13. Protecting health workers in high-conflict regions
14. Gender and Medical Education - Women in Surgery
15. Food Insecurity and Malnutrition

## Appendix 2

1. *Roughly* how much time did you spend working on your project independently?
  - Less than 30 minutes
  - 30 min - 1 hour
  - 1 hour - 1.5 hours
  - 1.5 hours - 2 hours
  - More than 2 hours
2. *Roughly* how much time did you spend working on your project in your group?
  - Less than 30 minutes
  - 30 min - 1 hour
  - 1 hour - 1.5 hours
  - 1.5 hours - 2 hours
  - More than 2 hours
3. *Roughly* how much time did you engage in cross cultural learning, friendship, regular conversation, etc.?
  - Less than 30 minutes
  - 30 min - 1 hour
  - 1 hour - 1.5 hours
  - 1.5 hours - 2 hours
  - More than 2 hours

Open Ended Short Questions

4. Have you gotten to know your partners better? Please describe any instances of growth, sharing, fun, difficulties, etc.
5. Rate the quality of your weekly interaction (1-10). Please explain your choice.
6. Please submit a brief, yet detailed synopsis of your weekly call, including discussion topics, progress, setbacks, fun activities, and other news. Detail is encouraged.
7. (Optional File Upload) Upload any photos or documents relevant to this week's session (if applicable).

## Appendix 3

https://docs.google.com/spreadsheets/d/1ux2y9M7hXGpajdj9S4TV1c7ZWlGnnnKq/edit#gid=1723545061

## Appendix 4

**Table 2:** GCCA Survey Results

Group	Access Time	**Internal Readiness**	Self Awareness	Risk Taking	Open Mindedness	Attentiveness to Diversity	**External Readiness**	Historical Perspective	Global Awareness	Intercultural Capability	Collaboration Across Cultures
UVA pre	**Group** **Mean**	**78.9**	**81.7**	**76.5**	**80.0**	**77.9**	**75.8**	**62.4**	**71.0**	**83.0**	**84.1**
UVA pre	***Standard Deviation***	*6.1*	*7.4*	*7.3*	*7.7*	*9.0*	*7.5*	*19.4*	*15.1*	*9.7*	*9.6*
UVA post	**Group** **Mean**	**78.4**	**78.2**	**73.9**	**81.6**	**79.6**	**74.4**	**72.8**	**63.3**	**84.2**	**77.0**
UVA post	***Standard Deviation***	*7.1*	*8.5*	*9.0*	*10.9*	*8.8*	*12.6*	*21.6*	*15.9*	*12.3*	*15.2*
UGHE pre	**Group** **Mean**	**64.1**	**67.6**	**61.9**	**65.7**	**61.8**	**56.9**	**36.2**	**50.7**	**72.2**	**64.9**
UGHE pre	***Standard Deviation***	*8.4*	*11.3*	*12.5*	*10.1*	*10.7*	*11.2*	*22.1*	*19.1*	*12.1*	*14.4*
UGHE post	**Group** **Mean**	**65.5**	**68.2**	**62.8**	**65.8**	**65.6**	**58.5**	**45.0**	**41.9**	**78.3**	**67.3**
UGHE post	***Standard Deviation***	*7.5*	*7.9*	*9.1*	*10.0*	*13.0*	*7.5*	*16.7*	*10.9*	*12.4*	*9.9*
